# 1564. Virological Suppression in People with HIV-1 (PWH) Receiving Dolutegravir/Lamivudine Was High and Similar across Age Groups despite Older PWH Having Increased Rates of Comorbidities and Polypharmacy (TANDEM Subgroup Analysis)

**DOI:** 10.1093/ofid/ofad500.1399

**Published:** 2023-11-27

**Authors:** Andrew P Brogan, Jihad Slim, Gustavo Verdier, Gavin Harper, Katie L Mycock, Hannah Wallis, Cynthia Donovan

**Affiliations:** ViiV Healthcare, San Diego, California; Saint Michael’s Medical Center, Newark, NJ, USA, Newark, New Jersey; ViiV Healthcare, Montréal, QC, Canada, Pointe-Claire, Quebec, Canada; Adelphi Real World, Bollington, England, United Kingdom; Adelphi Real World, Bollington, England, United Kingdom; Adelphi Real World, Bollington, England, United Kingdom; ViiV Healthcare, San Diego, California

## Abstract

**Background:**

Aging with HIV has unique challenges, including comorbidities and drug-drug interactions. TANDEM was a retrospective medical chart review conducted across 24 US sites. This subgroup analysis examines stable-switch PWH receiving dolutegravir/lamivudine by age.

**Methods:**

Eligible PWH were adults initiating dolutegravir/lamivudine prior to September 30, 2020, with ≥6 months of clinical follow-up (Figure 1). Stable-switch PWH were defined as having HIV-1 RNA < 50 copies/mL and on a stable antiretroviral regimen for ≥3 months upon dolutegravir/lamivudine initiation. Clinical characteristics, treatment history, and outcomes were analyzed for 3 age groups among dolutegravir/lamivudine stable-switch PWH. Analyses were descriptive.

**Figure:**
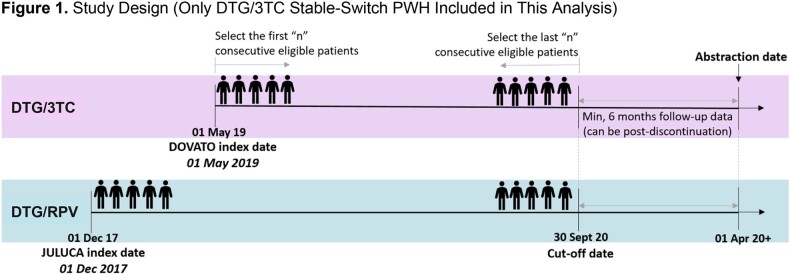

**Results:**

The number of dolutegravir/lamivudine stable-switch PWH in each age group was 86 (< 50 years), 106 (≥50 years), and 20 (≥65 years). A greater proportion of PWH in the older age groups were female sex at birth (14.0% < 50 years; 20.8% ≥50 years; 30.0% ≥65 years). More PWH in older age groups had reported comorbidity (12.8% < 50 years; 34.9% ≥50 years; 45.0% ≥65 years) and polypharmacy (5.8% < 50 years; 17.9% ≥50 years; 30.0% ≥65 years; Figure 2). Avoidance of long-term toxicities was the biggest driver for initiating dolutegravir/lamivudine in the older age groups (32.1% ≥50 years; 30.0% ≥65 years) while simplification/streamlining of treatment was the most common (27.9%) primary reason for PWH to switch to dolutegravir/lamivudine in the < 50 years group (Figure 3). For PWH < 50 years, 95.3% remained virologically suppressed for the study period. Similarly, 96.2% of PWH ≥50 years and 95.0% of PWH ≥65 years remained virologically suppressed for the study period (Figure 4). Three PWH with nucleoside/nucleotide reverse transcriptase inhibitor (NRTI) resistance at baseline remained suppressed.

**Figure:**
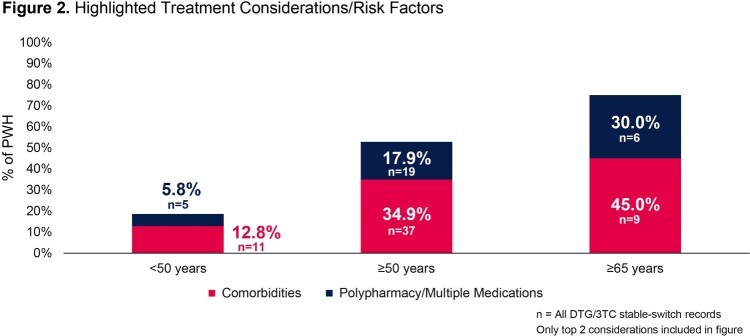

**Figure:**
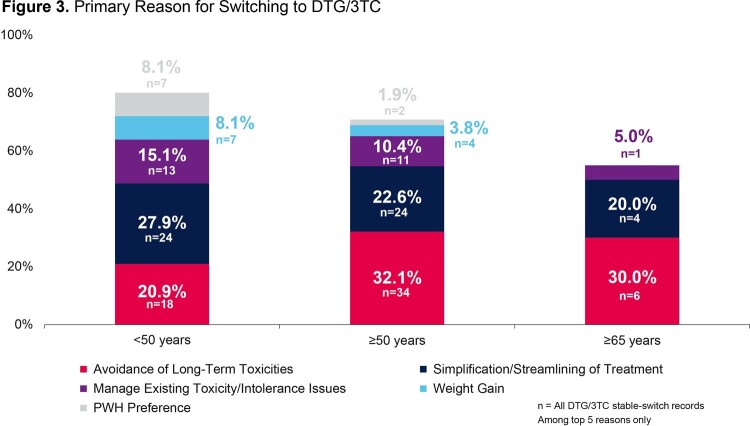

**Figure:**
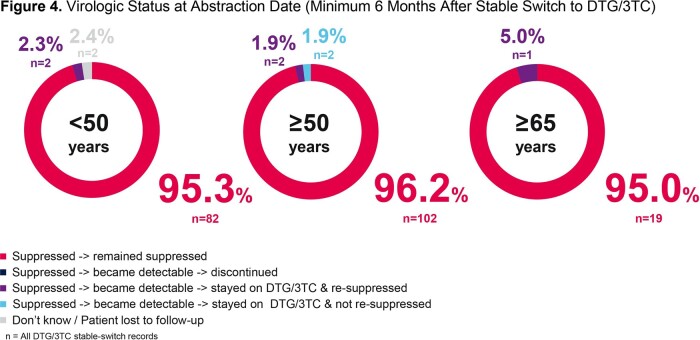

**Conclusion:**

Virological suppression rates were high and similar by age group among dolutegravir/lamivudine stable-switch PWH despite higher rates of comorbidities and polypharmacy in the older age groups. This real-world analysis is consistent with clinical study data supporting dolutegravir/lamivudine as an effective treatment strategy in older PWH.

**Disclosures:**

**Andrew P. Brogan, PhD**, ViiV Healthcare: Stocks/Bonds **Jihad Slim, MD, FACP**, ViiV Healthcare: Advisor/Consultant|ViiV Healthcare: Grant/Research Support **Gustavo Verdier, BSc, BPharm, MBA**, ViiV Healthcare: Stocks/Bonds **Gavin Harper, BA**, ViiV Healthcare: Grant/Research Support **Katie L. Mycock, MChem**, ViiV Healthcare: Grant/Research Support **Hannah Wallis, MS**, ViiV Healthcare: Grant/Research Support **Cynthia Donovan, PharmD**, ViiV Healthcare: Stocks/Bonds

